# Genetic strategies to investigate neuronal circuit properties using stem cell-derived neurons

**DOI:** 10.3389/fncel.2012.00059

**Published:** 2012-12-18

**Authors:** Isabella Garcia, Cynthia Kim, Benjamin R. Arenkiel

**Affiliations:** ^1^Program in Developmental Biology, Baylor College of MedicineHouston, TX, USA; ^2^Medical Scientist Training Program, Baylor College of MedicineHouston, TX, USA; ^3^Department of Molecular and Human Genetics, Baylor College of MedicineHouston, TX, USA; ^4^Jan and Dan Duncan Neurological Research Institute, Texas Children's HospitalHouston, TX, USA

**Keywords:** stem cell, synapse, circuit, neuron, optogenetic, transsynaptic, reprogramming

## Abstract

The mammalian brain is anatomically and functionally complex, and prone to diverse forms of injury and neuropathology. Scientists have long strived to develop cell replacement therapies to repair damaged and diseased nervous tissue. However, this goal has remained unrealized for various reasons, including nascent knowledge of neuronal development, the inability to track and manipulate transplanted cells within complex neuronal networks, and host graft rejection. Recent advances in embryonic stem cell (ESC) and induced pluripotent stem cell (iPSC) technology, alongside novel genetic strategies to mark and manipulate stem cell-derived neurons, now provide unprecedented opportunities to investigate complex neuronal circuits in both healthy and diseased brains. Here, we review current technologies aimed at generating and manipulating neurons derived from ESCs and iPSCs toward investigation and manipulation of complex neuronal circuits, ultimately leading to the design and development of novel cell-based therapeutic approaches.

## Introduction

**Figure 1:**
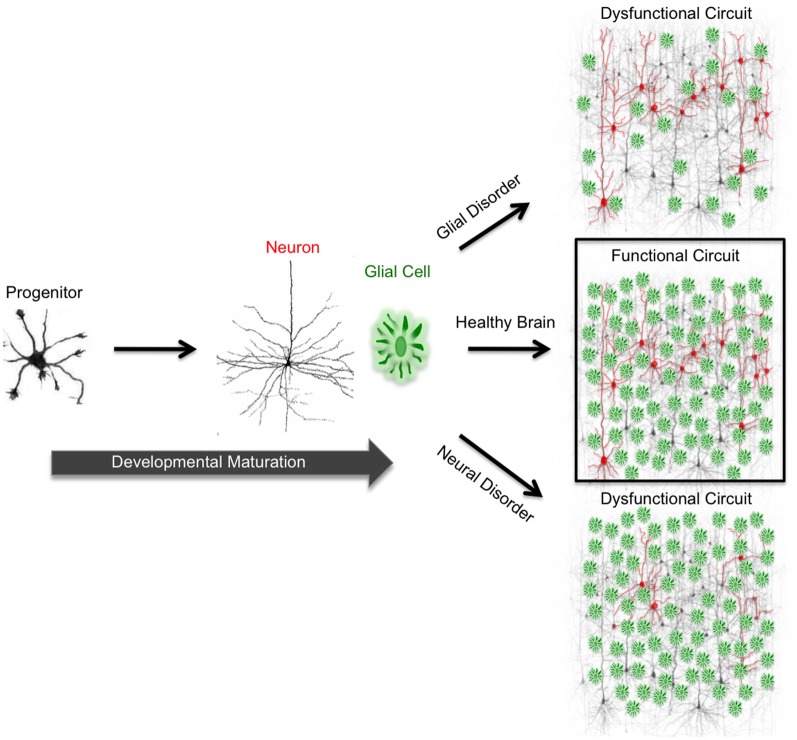
**Functional circuit formation stems from precise coordination between diverse neuronal and glial subtypes.** In the mammalian brain, neurons are connected to each other via synapses, forming complex neuronal circuits. The development and maturation of these circuits requires precise coordination between heterogeneous cell populations consisting of diverse neuron and glial subtypes. During development, neural progenitor cells give rise to neurons and glia. In neurodevelopmental and neurodegenerative disorders, proper neuron and glial interactions do not occur due to cellular malfunction. This malfunction impairs functional circuit formation and leads to circuit dysfunction. Impaired neuronal circuits are implicated in a wide variety of disorders such as epilepsy, Alzheimer's, Parkinson's, and Huntington's diseases.

Within the mammalian nervous system, billions of neurons are intricately interconnected via trillions of synapses. From this complex network of synaptic connectivity, specialized neural circuits emerge, forming the foundation for diverse physiological processes and behaviors. Due to the sheer complexity and heterogeneity of the cell and tissue types that contribute to neural circuits, the brain is prone to an array of developmental and neurodegenerative diseases, ranging from epilepsy (Noebels et al., 2012; Pun et al., 2012), mental illness (Liemburg et al., 2012a,b), Alzheimer's disease (Cao et al., 2012; Grienberger et al., 2012), Parkinson's disease (Armstrong et al., 2002; Rochester et al., 2012), and Huntington's disease (Mazzocchi-Jones et al., 2009; Niclis et al., 2009). Although highly heterogeneous in presentation, etiology, and affected cell populations, neurological disorders fundamentally involve the loss of proper circuit function (Figure 1) (Miller et al., 2011; Noutel et al., 2011; Ramamoorthi and Lin, 2011; Wang et al., 2011; Wesson et al., 2011; Ghiglieri et al., 2012). In recent times, pharmacological interventions have shown therapeutic promise toward the treatment of many neurological disorders (Kraft et al., 2009; Ballatore et al., 2012; Das et al., 2012; Fridhandler et al., 2012; Pedersen et al., 2012; Weaver et al., 2012). For example, several forms of epilepsy can be well controlled with pharmacology. Yet, in cases of severe neurodegenerative disorders, drug therapy has shown more variable results. This variability is compounded by the fact that medications are often initiated at late stages of disease when symptoms become clinically evident, providing limited, if any, symptomatic relief. Even in cases with promising drug therapy, one major limitation is that patients may become desensitized and thus require increasingly higher doses of medication until they reach the point of tolerance (Gancher et al., 1996). Further, even if a patient's symptoms are controlled with medication, prohibitive side effects may restrict the utility of pharmacological therapy. Finding the right therapeutic approach is therefore a challenge for many patients, especially when such limits of drug therapy have been reached. Neurosurgical options, including deep brain stimulation implants in Parkinson's disease patients, are increasingly offered to those whose symptoms are pharmacologically non-responsive, to promote activity of remaining functional neuronal tissue. However, these approaches can be accompanied by the general risks of invasive procedures, and their mechanisms of action remain to be characterized. In the emerging era of stem cell therapy, much hope lies in developing a less invasive, cell-based therapy that offers the unique ability to replace dysfunctional, damaged, or lost neurons. The promise of curative approaches for such progressive and devastating conditions as Huntington's disease, Alzheimer's disease, Parkinson's disease, and Amyotrophic Lateral Sclerosis has lured many patients to undergo alleged stem cell transplants from a range of medical providers, offering these procedures in absence of a critical mass of controlled clinical trials and detailed understanding of the mechanisms of a cell-based therapeutic approach to neurodegenerative diseases.

Recent efforts have focused on developing mechanistic insight into cell-based therapies (Alvarez Dolado and Broccoli, 2011; Dyson and Barker, 2011; Freed et al., 2011; Chen and Blurton-Jones, 2012; Kauhausen et al., 2012; Moviglia et al., 2012). Cellular approaches aimed at understanding and treating neurological diseases have ranged from the direct transplantation of embryonic, neuronal, and fetal stem cells (Bjorklund et al., 2002; Erdo et al., 2004; Hattiangady et al., 2007; Kauhausen et al., 2012; Moon et al., 2012) in rodent models, to the *in vitro* generation and investigation of induced pluripotent stem cells (iPSCs) and induced neural stem cells (iNSCs) from affected patients' tissues (Takahashi and Yamanaka, 2006; Takahashi et al., 2007; Han et al., 2012; Ring et al., 2012; Thier et al., 2012). Although induced stem cells have yielded valuable *in vitro* models of several neurological disorders (Camnasio et al., 2012; Israel et al., 2012; Ooi et al., 2012; Yagi et al., 2012), numerous studies have identified major challenges that have hindered transplantation efforts. Notable examples include teratoma formation (Bjorklund et al., 2002; Seminatore et al., 2010; Cunningham et al., 2012; Garcia et al., 2012), graft rejection (Krystkowiak et al., 2007), neuronal death (Nolte et al., 2008; Wang et al., 2012), and improper integration into pre-existing brain circuits (Kelly et al., 2007; Wang et al., 2012). In order to begin to harness the potential of stem cell therapy toward the treatment of neurological disorders, these issues must be addressed. Here, we review current literature regarding the generation of neurons from different stem cell populations and discuss their potential use for both *in vitro* studies and *in vivo* transplantation. We further provide an overview of current strategies to mark and manipulate neuronal activity in intact brain tissues, and discuss the interface between these genetic and cellular technologies to investigate circuit formation and function. Finally, we conclude by exploring the future of therapeutic interventions for damaged and diseased nervous systems using genetically modified stem cell-derived neurons.

## Generating neurons from embryonic stem cells

In the hopes of curing or providing therapeutic measures for damaged and diseased nervous tissue, significant interest has been placed in developing stem cell-based therapies for common neurological disorders (Babaei et al., 2012; Chen and Blurton-Jones, 2012; Lescaudron et al., 2012; Moon et al., 2012). Early attempts in animal models were aimed at transplanting pure populations of ESCs directly into damaged or diseased brain tissue (Deacon et al., 1998; Bjorklund et al., 2002; Erdo et al., 2004), with the intent of providing a source of renewable cells capable of functionally integrating into existing circuits. This notion was supported by optimism that naive stem cells would respond to cues from the surrounding tissue and ultimately differentiate and function as mature neurons with appropriate synaptic connections. However, many obstacles have hindered this approach. First, transplantation of pluripotent stem cells into the brains of animals can lead to restrictively high incidence of teratomas (Bjorklund et al., 2002; Erdo et al., 2004); in some cases, 25% or more of all grafts can result in undifferentiated brain tumors (Garcia et al., 2012). In attempt to avoid teratoma formation, efforts next turned toward transplantation of adult and fetal neural stem cells (NSCs) (Fainstein et al., 2012; Moon et al., 2012; Muneton-Gomez et al., 2012). With putative lineage restriction, NSCs were considered to have potential as a renewable source of neuronal and glial subtypes, without the attendant risk of teratoma formation. However, in lieu of generalized teratoma formation, transplanted NSCs have been observed to produce neural lineage-restricted brain tumors, such as medulloblastomas and gliomas in animal models (Swartling et al., 2012). Alongside these challenges other obstacles have surfaced, including graft rejection (Capetian et al., 2011; Chen et al., 2011). While cell transplantation can be straightforward, the procedure may activate host immune responses, which can result in rejection of transplanted cells prior to circuit integration. To circumvent this, immunosuppressive drugs are required during and after cell transplantation (Leveque et al., 2011; Hovakimyan et al., 2012). Unfortunately, immunosuppressants do not completely eliminate graft rejection, and they invite susceptibility to various types of opportunistic infections (Kasper et al., 2011; Pustavoitau et al., 2011; Dhar et al., 2012).

**Figure 2:**
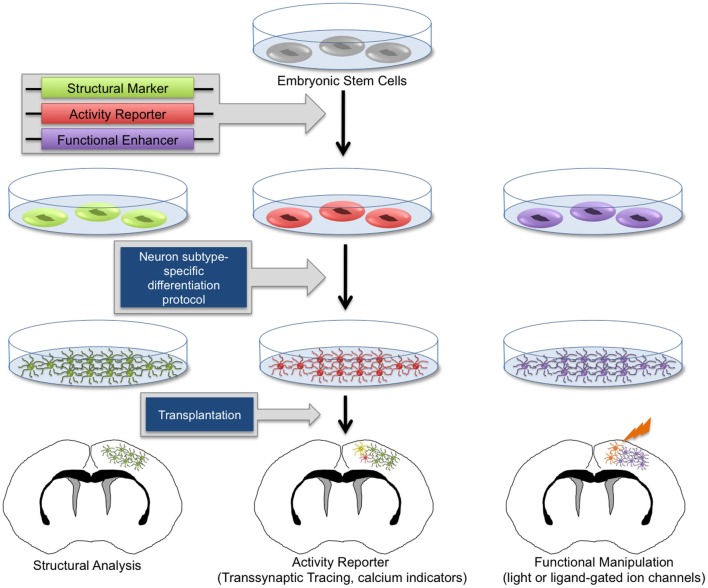
**Genetic manipulation of embryonic stem cell-derived neurons.** Human and mouse embryonic stem cells (ESCs) can be routinely cultured *in vitro*. Through genetic manipulations, stem cells can be endowed with structural reporters such as fluorescent markers, activity reporters including transsynaptic tracing machinery and calcium indicators, as well as functional enhancers such as light-responsive proteins and exogenous ligand-activated ion channels. Upon neuron subtype-specific differentiation, modified ESCs will give rise to heterogeneous neuronal and glial subtypes. *In vitro*-generated neurons can be transplanted into healthy and diseased brain tissue. With this technology, it is possible to perform morphological analysis, transsynaptic tracing, and activity measurements and manipulation of transplanted neurons, allowing the functional investigation of neuronal circuit integration in the brain.

Significant efforts have since been made to develop *in vitro* differentiation protocols to program stem cells into distinct neuronal lineages, thus avoiding unrestricted clonal expansion and tumor formation prior to terminal differentiation (Caiazzo et al., 2011; Chung et al., 2011; Jing et al., 2011; Juliandi et al., 2012; Kirkeby et al., 2012). It is now possible to routinely generate high numbers of ESC-derived neurons of various subtypes *in vitro* (Bibel et al., 2007; Parsons et al., 2011; Garcia et al., 2012; Kirkeby et al., 2012; Moon et al., 2012; Salti et al., 2012). Molecular marker analysis and electrophysiological characterizations have demonstrated that these neurons appropriately differentiate *in vitro* and exhibit patterns of action potentials and neurochemical profiles characteristic of neurons found in intact brain tissue (Bissonnette et al., 2011; Caiazzo et al., 2011; Cho et al., 2011; Garcia et al., 2012). Using specialized protocols, heterogeneous populations of NSCs, as well as GABAergic, glutamatergic, dopaminergic, and various other neuronal and glial subpopulations can be obtained at high quantities. Importantly, using these *in vitro*-generated neuronal subpopulations, it is possible to specifically target affected cell populations in diverse disease models. Furthermore, these differentiated cellular subtypes provide a valuable resource for *in vitro* studies. With renewable sources of diverse neuronal lineages, numerous studies have implemented transplantation methods to attempt treatment of specific neurological disorders. In some cases, therapeutic effects have been observed (Song et al., 2007; Chung et al., 2011; Kriks et al., 2011). For example, improved behavioral outcomes in animal models of Parkinson's disease and other movement disorders have been observed following stem cell-based therapies (Song et al., 2007; Chung et al., 2011; Kriks et al., 2011), and transplantation of GABAergic neurons have shown modest decreases in seizure activity in epilepsy models (Castillo et al., 2008; Maisano et al., 2009). However, it remains unknown whether observed therapeutic effects are evidence of functional restoration by engrafted cells or are the result of stromal neuroprotective effects, including improved vascularization and secretion of neurotrophic factors. Therefore, it has become a priority to investigate the potential for transplanted progenitor cells to properly differentiate, synaptically mature, and appropriately integrate within functional neural circuits *in vivo*. Toward this effort, numerous methodologies have emerged to differentiate stem cells into neuronal lineages and genetically engineer them to mark and manipulate their patterns of synaptic connectivity (Figure 2).

## Generating neural stem cells from somatic cell types

To circumvent host stem cell graft rejection, emphasis has been placed on developing methods to genetically reprogram adult somatic cells to a state of pluripotency, resembling embryonic stem cells (ESCs). These iPSCs can be differentiated into neuronal subtypes *in vitro* and autologously transplanted back into the donor brain. Because these cells are patient-derived, they are thought less likely to be rejected by the host immune system, leading to improved graft survival. The first successful method of cellular reprogramming published by Takahashi and Yamanaka, demonstrated that somatic cells from both mice (Takahashi and Yamanaka, 2006) and humans (Takahashi et al., 2007) can be manipulated to adopt a pluripotent state. This discovery received the Nobel Prize in Physiology or Medicine in 2012. Cellular reprogramming is accomplished by ectopic expression of transcription factors in somatic cells. When cultured appropriately, these cells further produce lineages derived from any of the three embryonic germ layers (Vierbuchen et al., 2010; Han et al., 2012; Ring et al., 2012; Thier et al., 2012). Notably, these studies demonstrate effective generation of ectodermal-like neuronal precursors with capacity to mature into functional neurons capable of generating specific patterns of action potentials *in vitro*. Techniques have since advanced to facilitate reprogramming from a variety of starting cell types, including fibroblasts (Pfisterer et al., 2011; Ring et al., 2012), keratinocytes (Petit et al., 2012), and blood cells (Ma et al., 2011). Additionally, methods have been devised to bypass the pluripotent state, thereby directly generating iNSCs (Hanna et al., 2007; Denham and Dottori, 2011; Pfisterer et al., 2011; Seminatore et al., 2011; Yusa et al., 2011; Han et al., 2012; Ring et al., 2012; Thier et al., 2012). Bypassing the pluripotent state allows for direct generation of neuronal subtypes, minimizing risk of teratoma formation. Reprogramming technology thus provides an elegant means to generate relatively inaccessible or growth-restricted cell lineages from readily obtained tissue samples, with virtually unlimited potential for both basic research and clinical application. One can perhaps envision a near future where biomarkers are used to identify neurological disease patients prior to symptom onset, and where the patient's own somatic cells can be used to validate and study these disorders without having to obtain neural biopsies via invasive measures.

However, similar to difficulties with ESC-derived neurons, efficacy of induced stem cell transplants in animal models can be complicated by tumorigenic transformation (Yamanaka, 2009; Fong et al., 2010) resulting from the genomic integration or sustained expression of reprogramming factors (Okita et al., 2008). Thus, there remains a need for more “factor-free” reprogramming technologies to eliminate oncogenic potential. Moreover, although studies are now beginning to evaluate *in vitro* connectivity of grafted neural cells with host tissue (Tonnesen et al., 2011), the long-term maintenance of these connections and the survival potential for *in vivo* transplants remain unknown. With the development of a more extensive genetic and biological toolset, iNSC technology is poised to overcome such limitations. Genetic manipulation and transplantation of stem cell-derived neurons into live animals will provide a better understanding of different measures of synaptic structure and/or function in the intact brain. Detailed investigations of neuronal organization promise to advance our working knowledge of circuit architecture and operation in both healthy and diseased brain tissue with ever-greater resolution.

## Viral-based approaches to investigate neuronal connectivity

In order to properly generate desired neuronal subtypes and target areas for transplantation studies, it is necessary to reveal the precise patterns of cellular connectivity in particular brain regions and in disease models. Classical studies have applied anatomical methods using histology, light microscopy, and ultrastructural analysis to label and identify neurons that make synaptic connections (Stevens et al., 1980; Anderson et al., 1994; Ahmed et al., 1997; Briggman and Denk, 2006; Micheva and Smith, 2007). Structural studies provide great insight into synaptic connectivity, but without additional functional analysis, these connections should perhaps be considered “passive.” To further characterize passive connectivity, a number of tracing methods have been implemented, including fluorogold retrograde tracers (Gomez-Nieto et al., 2008; Rossetti et al., 2012), biotinylated dextrans (Shehab et al., 2005; Rossetti et al., 2012), wheat germ agglutinin (Louis et al., 2010), cholera toxin conjugated dyes (Angelucci et al., 1996; Miyashita and Rockland, 2007), fluorescent microspheres (Apps and Ruigrok, 2007; Neely et al., 2009), and lipophilic dyes (Bruce et al., 1997; Makarenko et al., 2000). However, a major limitation to these methods is the gradual signal decline with distance or degree of labeled projections. Additionally, indiscriminate cell-to-cell spread can occur between contacting cells in close proximity, obscuring the interpretation of functional connectivity. As such, these classical technologies label connected neurons anatomically, but do not reveal true functional connections.

To elucidate networks between synaptically-coupled partners more precisely, novel technologies using neurotropic viruses have been established (Callaway, 2008; Ugolini, 2010, 2011; Arenkiel, 2011; Arenkiel et al., 2011). Two prominent subtypes of viruses used for transneuronal tracing include rabies virus (RV) and herpes virus (Callaway, 2008; Ugolini, 2010). Both infect cells via retrograde particle transfer, allowing for the identification of neurons presynaptic to an infected source cell. In order to determine the precise identity of synaptic partners, fluorescent proteins have been engineered into the herpes and rabies viral genomes (Kuypers and Ugolini, 1990; Wickersham et al., 2007a; Callaway, 2008), allowing synaptically connected cells to be visualized via detection of viral transfer using fluorescence microscopy. Because these viral particles are self-replicating, their signal is sustained over time and distance, and all synapses are labeled with similar efficiency. The two most common herpes strains used for viral tracing include herpes simplex virus 1 (HSV-1) (Lilley et al., 2001) and pseudorabies virus (PRV) (Enquist, 2002). Both strains are potent neuronal tracers and mark synaptically connected neurons with very high efficiency. However, a major limitation of the use of herpes virus for synaptic tracing studies is its polysynaptic spread (Callaway, 2008; Ugolini, 2010). Herpes virus moves across synapses very quickly, rapidly infecting brain tissue and making it difficult to dissect precise synaptic connections. Additionally, herpes virus integrates into cellular genomes and promptly becomes toxic to the infected animal, leading to death within days (Ugolini, 2010). To overcome these limitations, other transsynaptic viral vectors have been engineered. Like HSV, RV spreads to synaptically connected neurons in a retrograde manner. But because its genome consists of negative strand RNA, RV does not integrate into cellular DNA and is less toxic to infected neurons (Callaway, 2008). Infected cells remain healthy for up to two weeks, allowing for the functional investigation of synaptic partners.

Wickersham et al. have demonstrated that genetically altered RV can be used for precise identification of synaptic partners (Wickersham et al., 2007a). To achieve this, the rabies glycoprotein G gene, which encodes the viral capsid and enables viral assembly and spread is deleted and replaced with the gene for enhanced green fluorescent protein (EGFP). These engineered RV particles infect neurons with high efficiency and use the host cellular machinery to replicate and produce high amounts of EGFP, without generating infectious virions. Because the viral genome is engineered without glycoprotein G, only primarily infected neurons are labeled (Wickersham et al., 2007a). To optimize engineered RV for transsynaptic circuit tracing, Wickersham et al., “pseudotyped” the viral particles with the foreign coat protein EnvA from the avian sarcoma leukosis virus (Wickersham et al., 2007b). EnvA recognizes the TVA receptor that is naturally only found on cell membranes of certain avian species and is not normally present on the mammalian neuronal membrane. By introducing cDNA encoding both the rabies G glycoprotein and TVA receptor, together with a fluorescent marker such as tdTomato for identification purposes, targeted neurons can be infected by the pseudotyped RV, which also encodes EGFP. Additionally, when exogenous glycoprotein G is provided *in trans*, the virus is mobilized to replicate, self-assemble, and carry out a single retrograde jump to its presynaptic inputs. Because presynaptic partners do not express glycoprotein G, and no further glycoprotein G is provided, viral tracing comes to a halt, marking monosynaptically connected input neurons (Wickersham et al., 2007b). Such tracing methods have been extremely powerful to deduce local patterns of neuronal connectivity (Arenkiel et al., 2011; Miyamichi et al., 2011).

**Figure 3:**
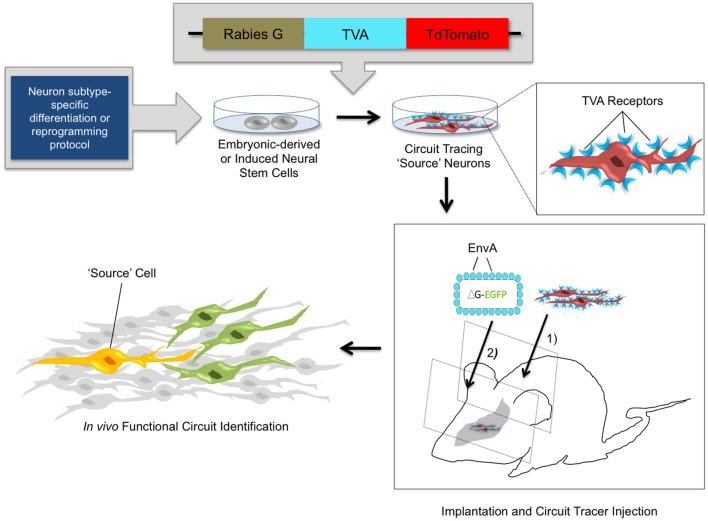
***In vivo* circuit analysis with derived or reprogrammed neurons harboring viral circuit tracing elements.** Using neuron subtype-specific differentiation or reprogramming protocols, a specialized population of neural stem cells can be obtained *in vitro*. Electroporation or stable transfection with a transsynaptic tracing vector provides the rabies G coat protein and the TVA receptor, which are components of the monosynaptic circuit tracing process. Incorporation of the viral tracing vector into the stem cell genome transforms these cells into “source” cells for circuit tracing. These cells can be implanted *in vivo* and identified by expression of the tdTomato fluorescent reporter. After a defined time period of maturation of the implanted cells, pseudotyped rabies virus is injected. The virus specifically targets TVA-expressing source cells and marks them with the EGFP fluorescent reporter. The source cells thus fluorescence both red and green, indicated by a yellow color. The circuit tracing system allows a single retrograde jump of virions carrying the EGFP reporter from the source cell to presynaptic neurons. This identifies synaptic connections and functional circuits formed by integrated neural stem cells with high specificity.

Recently, stable mouse ESC lines have been engineered to harbor transsynaptic tracing elements (glycoprotein G, TVA, and a tdTomato reporter) (Garcia et al., 2012). These cells can be differentiated with high efficiency into diverse neuronal lineages as verified via molecular marker expression and electrophysiological analysis. When introduced into slice cultures *in vitro*, or into intact brain tissue *in vivo*, the transplanted cells can be identified by fluorescent reporter expression and subsequently infected with pseudotyped RV to act as source cells for circuit tracing studies (Figure 3). In the past, studies with NSC transplants have used the expression of fluorescent reporters (Chang et al., 2012; Steinbeck et al., 2012), magnetic resonance imaging, positron emission tomography (Daadi et al., 2009; Tang et al., 2011; Chang et al., 2012), and phenotypic rescue (Yang et al., 2008; Nagai et al., 2010; Chung et al., 2011; Zhu et al., 2011) to verify the integration of stem cell-derived neurons into brain circuits. However, whether transplanted cells forge functional connections within existing circuitry has remained difficult to ascertain. Through new genetic tracing technologies, it is now feasible to elucidate local networks of synaptic connectivity and to determine the cast of presynaptic contacts that form onto transplanted stem-cell derived neurons. With the knowledge obtained from neuronal tracing studies in healthy and diseased brains, combined with genetic strategies for neuronal activity manipulations, it is possible to genetically target healthy and diseased neuronal circuits for precise labeling and/or control of neuronal firing (Figure 2).

## Genetic approaches to manipulate neural circuit activity

The desire to use cell-based therapeutic approaches toward the treatment of damaged or diseased nervous tissue is rapidly gaining popularity. However, prospects for these approaches depend both on the ability to guide selective programs of neuronal differentiation, and to engineer cells whose output can be precisely controlled. In the adult brain, it has been established that diverse forms of activity govern the integration and survival of new neurons (Nilsson et al., 1999; van Praag et al., 1999; Rochefort et al., 2002; Leuner et al., 2004; Rochefort and Lledo, 2005; Yamaguchi and Mori, 2005). Through application of novel genetic tools, it is now possible to artificially “induce” or “silence” activity in brain circuits using light (Nagel et al., 2005; Arenkiel et al., 2007; Zhang et al., 2007a,b; Chow et al., 2010; Deisseroth, 2010; Zhang et al., 2010; Tonnesen et al., 2011; Madisen et al., 2012), or exogenous chemical ligands (Tan et al., 2006; Lerchner et al., 2007; Arenkiel et al., 2008; Conklin et al., 2008; Drenan et al., 2008; Wehr et al., 2009). Furthermore, genetically encoded calcium indicators provide a method for functional readout in neurons (Pologruto et al., 2004; Mao et al., 2008; Tian et al., 2009; Akerboom et al., 2012; Chen et al., 2012). This capability is especially valuable for cell-based circuit and tissue repair, where integration and functional control may be desired. Early efforts toward manipulating neuronal activity in select cell types have used exogenous ligands not normally present in the brain (Arenkiel et al., 2008; Drenan et al., 2008; Wulff and Arenkiel, 2012). Chemical genetic technologies have demonstrated both the activation (Arenkiel et al., 2007; Conklin et al., 2008; Drenan et al., 2008) and silencing (Tan et al., 2006; Lerchner et al., 2007; Wehr et al., 2009) of select neuronal cell types in intact brain tissue both *in vitro* and *in vivo*. For example, the transgenic overexpression of ligand-gated ion channels (LGIC) in neuronal subtypes allows cellular activation upon exogenous ligand administration (Drenan et al., 2008; Magnus et al., 2011). Examples of chemical genetic tools for neuronal activation include expression of modified opiate receptors (Coward et al., 1998), custom G_q_-protein coupled receptors with high sensitivity for synthetic ligands (DREADD hM3Dq) (Armbruster et al., 2007), hyperdopaminergic activity via ectopic acetylcholine receptor activation (Drenan et al., 2008), as well as expression of the transient receptor potential cation channel subfamily V member 1 (TRPV1), which is normally expressed in nociceptive peripheral neurons and can be potently activated by administration of capsaicin (Arenkiel et al., 2008). Alongside this, several chemical genetic tools have also been developed to artificially silence neuronal activation, and include ectopic expression of a glutamate-gated chloride channel (GluCl) from *Caenorhabditis elegans*, which is activated by the antihelminthic drug ivermectin (Slimko et al., 2002; Lerchner et al., 2007), G_i_-coupled receptors with high sentitivity for synthetic ligands (DREADD hM4Di) (Arenkiel et al., 2007), as well as expression of the *Drosophila* allatostatin receptor (AlstR) which induces G_i_-coupled silencing in the presence of the insect peptide allatostatin (Birgul et al., 1999). Using chemical genetics, it becomes possible to precisely control neuronal output with high specificity. However, the use of exogenous ligands in living organisms poses several challenges. For example, it is necessary to consider the intrinsic properties of the ligand itself, as well as potential homeostatic mechanisms present in living tissues. Notably, engineered ligands must be inert and able to cross the blood-brain barrier. Further, ligands should be non-toxic to the animal and rapidly degraded in order to allow for precise time-dependent control of neuronal manipulation. Because of the challenges posed by chemical genetic technologies, researchers have also implemented optical genetic methods for controlling neuronal activity with light (Nagel et al., 2005; Arenkiel et al., 2007; Chow et al., 2010; Deisseroth, 2010).

Optogenetic approaches allow for fast and precise control of neuronal activity using light-activated ion channels via a spectrum of different wavelengths (Zhang et al., 2007a,b, 2010). The most popular light-activated ion channels used for neuronal depolarization are the channelrhodopsins, originally identified in the green algae *Chlamydomonas reinhardtii* (Nagel et al., 2005; Deisseroth, 2010). In response to blue light (480 nm), channelrhodopsin-2 (ChR2)-expressing neurons rapidly influx cations, which depolarize the cell membrane, leading to firing of action potentials. ChR2-assisted circuit mapping (CRACM) has allowed precise dissection of neuronal circuits by stimulating and activating ChR2-expressing neurons while recording electrophysiological responses in postsynaptic neurons (Petreanu et al., 2007). Alternatively, action potential inhibition can be achieved via light-activated hyperpolarizing channels. This may be accomplished by either light-directed anion influx, or proton efflux. Halorhodopsin (NpHR) is a light-driven chloride pump that is activated with yellow-green light (570 nm) to drive chloride ions into cells, whereas archaerhodopsin actively drives protons out of the cell upon illumination with yellow-green light (Chow et al., 2010; Madisen et al., 2012). *In vitro* differentiated neurons harboring ChR2 and NpHR have recently revealed important information regarding the functional integration of intrastriatal grafts in a Parkinson's disease model (Tonnesen et al., 2011).

Additionally, genetically encoded calcium indicators such as GCaMP allow for functional readout of manipulated neurons (Pologruto et al., 2004; Mao et al., 2008; Tian et al., 2009; Akerboom et al., 2012; Chen et al., 2012). GCaMP is a modified GFP fused to calmodulin. Upon calcium influx, such as in the case of depolarization, GCaMP undergoes a conformational change, leading to increased fluorescence intensity. In the absence of calcium influx, the GCaMP-expressing cells fluoresce dimly.

**Figure 4:**
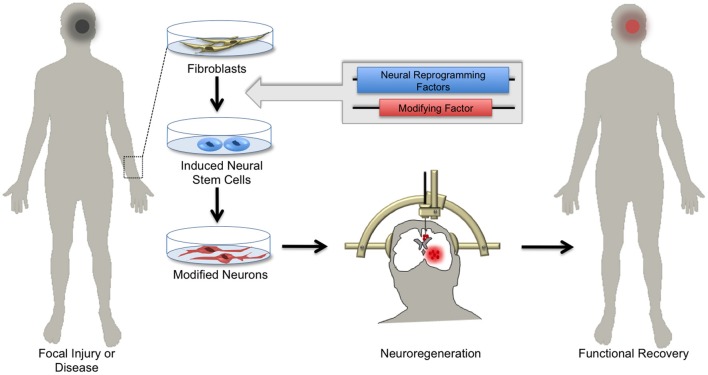
**Hopes for using induced neuronal stem cells for regenerative medicine.** Skin biopsies from patients with focal neurological injury or neurodegenerative disease are a ready source of fibroblasts. Using the approach of cellular reprogramming and subsequent genetic manipulations, patient-derived fibroblasts can be differentiated into induced neural stem cells (iNSCs) harboring functional elements. Depending on the patient's needs, iNSCs can be differentiated *in vitro* into specialized neuronal subtypes. Patient-derived neurons can then be transplanted by local injection into areas of diseased or damaged brain tissue. Upon integration, these cells regenerate parenchymal tissue, replacing dysfunctional cells and ultimately supporting functional recovery.

The emerging array of genetic tools for precise manipulation of neuronal activity gives hope to transitioning stem cell therapy from the conceptual to the clinical realm. For example, it may soon be possible to generate NSCs expressing detectable markers and/or activity-induced ion channels from patients with damaged or diseased nervous tissue and transplant these cells back into the affected patient (Figure 4). Such genetic strategies might enable monitoring of the integration and survival of transplant-derived neurons, and perhaps through controlled activity manipulations result in symptomatic relief in select neurological disorders.

## Concluding remarks

Advances in stem cell research combined with powerful genetic technologies now allow unprecedented levels of investigation and manipulation of complex neuronal circuits. Previous efforts to implement cell-based therapies have been hindered by tumorigenesis, graft rejection, cell death, lack of circuit integration, and the inability to follow grafted cells *in vivo*. Scientific advances are beginning to tackle these challenges. Further, transsynaptic tracing has enabled high resolution dissection of neuronal circuits, which has begun to reveal insights into the molecular mechanisms that guide synapse formation and circuit integration in the living brain. Genetic approaches to manipulate neural circuit activity have allowed for the selective “activation” and “silencing” of discrete neuronal subtypes. This knowledge, combined with the ability to generate high numbers of neurons *in vitro*, promises to yield significant advances in cell therapy. In the future, it might be possible to manipulate injured and/or diseased brain circuits with artificially grafted cells, allowing for sustained symptomatic relief in a range of neurological disorder and neuronal injury models.

### Conflict of interest statement

The authors declare that the research was conducted in the absence of any commercial or financial relationships that could be construed as a potential conflict of interest.
